# What to do with anti-science influencers?

**DOI:** 10.31744/einstein_journal/2026ED2641

**Published:** 2026-06-08

**Authors:** Jacyr Pasternak

**Affiliations:** 1 Hospital Israelita Albert Einstein São Paulo SP Brazil Hospital Israelita Albert Einstein, São Paulo, SP, Brazil.

This is not a new phenomenon. Scientific medical information has always competed with people promoting both new and old theories and remedies lacking a solid evidence base. In the past, this was part of the social milieu, and did not raise many concerns. However, social networks have changed this dynamic, allowing unscientific information to spread wildly.^(1)^Medicine is not easy to understand: complicated biological information is used, and evidence-based medicine requires physicians to read and understand complex statistics and evaluate clinical trials. Sometimes what is presented in papers is not the whole story. Skepticism is part of a good physician’s mental armamentarium.

The skeptical attitude is the opposite of every miracle cure being promoted on social networks, newspapers and TV. They present their nostrums as so effective that it would be unethical to conduct a controlled study. They are based on testimonials and have obviously never heard of placebos. Real scientists have a duty to engage with the public and educate people. They should use all possible media. American scientists hold meetings on the Washington mall, facing the Capitol, in defense of science. I do not think we should do the same at the *Praça dos Tres Poderes*, in Brasilia: our Congress is a lost cause. We should instead promote real science in all possible spaces.^(2)^As a matter of fact, most scientists and medical investigators are not good at explaining in simple terms what they do, and they fear criticizing some of the snake oil sellers because in the realm of social networks vicious personal attacks are common. Our journal publishes high-quality papers, after peer review. We are not the place where scientific knowledge in the health sciences is presented to the public. This is not the appropriate forum to go after the quacks and charlatans that infest social networks and increasingly, occupy prominent positions, as seen with many vaccine deniers today in leading roles in the US.^(3)^ What we would like to emphasize is that scientists, real ones, have an ethical duty to confront quacks and extremists. They should go to social networks, newspapers, radio and TV. Not everybody, for sure: you have to be a communicator or learn the ropes. You need to develop really thick skin. It’s not for everyone, but someone must do it. At least tell your circle of contacts and your family how to distinguish good information from misinformation and hallucinations.
